# Fabrication of Second Generation Smarter PLGA Based Nanocrystal Carriers for Improvement of Drug Delivery and Therapeutic Efficacy of Gliclazide in Type-2 Diabetes Rat Model

**DOI:** 10.1038/s41598-019-53996-4

**Published:** 2019-11-22

**Authors:** Bibhu Prasad Panda, Rachna Krishnamoorthy, Subrat Kumar Bhattamisra, Naveen Kumar Hawala Shivashekaregowda, Low Bin Seng, Sujata Patnaik

**Affiliations:** 1 Department of Pharmaceutical Technology, School of Pharmacy, Taylor’s University, Lakeside Campus, No 1, Jalan Taylor’s, 47500 Subang Jaya, Selangor Malaysia; 2 Department of Life Sciences, School of Pharmacy, International Medical University, Kuala Lumpur, 57000 Malaysia; 3 School of Pharmacy, Taylor’s University, Lakeside Campus, No 1, Jalan Taylor’s, 47500 Subang Jaya, Selangor Malaysia; 4 School of Medicine, Taylor’s University, Lakeside Campus, No 1, Jalan Taylor’s, 47500 Subang Jaya, Selangor Malaysia; 5 University College of Pharmaceutical Sciences, Kakatiya University, Warangal, Telangana India

**Keywords:** Type 2 diabetes, Drug delivery

## Abstract

Drug delivery and therapeutic challenges of gliclazide, a BCS class II drug used in type 2 diabetes mellitus (T2DM) can be overcome by exploring smarter carriers of second-generation nanocrystals (SGNCs). A combined method of emulsion diffusion, high-pressure homogenization and solvent evaporation method were employed in the preparation of gliclazide loaded poly (D, L-lactide-co-glycolide) (PLGA) SGNCs. Taguchi experimental design was adopted in fabrication of Gliclazide SGNc using Gliclazide -PLGA ratio at 1:0.5, 1:0.75, 1:1 with stabilizer (Poloxamer-188, PEG 4000, HPMC E15 at 0.5, 0.75, 1% w/v). The formulated gliclazide of SGNCs were investigated for physicochemical properties, *in vitro* drug release, and *in vivo* performance studies using type-2 diabetes rat model. The formulation (SGNCF1) with Drug: PLGA 1: 0.5 ratio with 0.5% w/v Poloxamer-188 produced optimized gliclazide SGNCs. SGNCF1 showed spherical shape, small particle size (106.3 ± 2.69 nm), good zeta potential (−18.2 ± 1.30 mV), small PDI (0.222 ± 0.104) and high entrapment efficiency (86.27 ± 0.222%). The solubility, dissolution rate and bioavailability of gliclazide SGNCs were significantly improved compared to pure gliclazide. The findings emphasize gliclazide SGNCs produce faster release initially, followed by delayed release with improved bioavailability, facilitate efficient delivery of gliclazide in T2DM with better therapeutic effect.

## Introduction

Type 2 diabetes mellitus (T2DM), is a non-communicable metabolic disorder characterized by high blood glucose, insulin resistance and relative insulin deficiency in the body. T2DM causes significant mortality and morbidity globally, which draws considerable attention from the public, policymakers and public health care providers for its mitigation and treatment^1,2^. According to the International Diabetes Federation (IDF), the global statistical data projected that 592 million people may have diabetes by the year 2035 and proportionally T2DM will increase in every country of the globe^3^. The major risk factors and causes of the epidemic of T2DM are obesity, aging, dietary practices, physical inactivity, urbanization, and a sedentary lifestyle^4,5^.

At present, the available hypoglycemic therapeutic agents are not successful in chronic treatment and management of T2DM due to failure in drug delivery, severe adverse effects and patient noncompliance associated with therapy management. There is a high demand for a holistic strategy, which focuses on prevention and intervention of a novel therapeutic approach in drug delivery to fight the menace of an inexorable T2DM^1,6^. Oral hypoglycemic drugs commonly prescribed in T2DM management are sulphonylureas, meglitinides, and thiazolidinediones therapeutic categories, belong to Biopharmaceutical Classification System (BCS) type II compounds with low solubility and high permeability. Poor aqueous solubility of BCS class II oral antidiabetics produces variable bioavailability, high fasted/fed state variation, retarded onset of therapeutic action, irreproducible therapeutic response and need a large amount of oral dose for the administration of hypoglycemic drugs^7–9^.

Gliclazide, a BCS class II drug, belongs to sulfonylurea group, clinically known to treat T2DM with its effectiveness and safety, is selected as a model drug for the study. Gliclazide, as a daily oral treatment for T2DM is known to stimulate insulin secretion by interacting with specific receptors on pancreatic β-cells, leads to a gradual improvement of glycaemic control^10–12^. The recommended dose for T2DM of gliclazide ranges from 40 mg to 320 mg daily. Currently, gliclazide tablets are available in the market in conventional immediate release or modified release dosage form to maintain normal plasma glucose levels. Single oral dose gliclazide shows, poor aqueous solubility, low and variable bioavailability, irreproducible therapeutic response and needs a large amount of oral dose for the administration of gliclazide due to low solubility in the stomach that varies the absorption in the intestine^13–15^. At present, available gliclazide dosage forms failed to meet the typical physiological goal such as basic needs between meals and during the night, which demands a faster drug release followed by prolonged drug release profile over an extended period to maintain constant plasma glucose level^16–18^. The literature review and its critical analysis on gliclazide, oral hypoglycemic BCS class II drug established that there is a huge demand for design and development of a novel delivery system, which counteracts drug delivery challenges, good bioavailability, established with good glycemic control with low therapeutic dose and better patient compliance^19–21^.

Research on solubility and drug delivery challenges of BCS class II drugs demonstrate that nanonization and functionalization of the drug into second-generation smarter nanocrystals is an ideal approach to meet these challenges. First generation nanocrystals are carrier-free colloidal delivery systems with nanosized or nanoscopic drug crystals with a mean particle size typically in the nanometer range, between 10–800 nm subject to appropriate nanosizing methods^22,23^. Classically, first generation nanocrystals were developed with a minimum amount of stabilizer by a single step, bottom-up technologies (precipitation methods) or top-down technologies (high-pressure homogenization)^24–26^. The recent advancement of nanocrystal technology is the second-generation nanocrystals (SGNCs) which is a colloidal delivery system, possesses a specialized carrier developed by a variety of specialized pre-combinative treatment strategies followed by a high-pressure homogenization technique^27,28^. The formulation strategies used in design, development, and delivery of polymeric SGNCs are simple, convenient, reliable and reproducible, which display remarkable improvement in solubility and bioavailability with a low therapeutic dose of BCS Class II drug. Novel possibilities of SGNCs application was explored and expedited by using poly (D, L-lactide-co-glycolide) PLGA based polymeric nanocrystal composite system as a modified delayed drug delivery carrier for BCS class II, oral hypoglycemic drug^1,29,30^. PLGA is a biodegradable and biocompatible polymer, most commonly and successfully used as a polymeric nanoparticulate drug delivery system for many therapeutic agents^31,32^. PLGA polymer biodegradation time varies from days to months depending on its molecular weight and ratio of lactic-glycolide copolymers composition. PLGA, which functions as a protective polymer, forms a surface grafted polymeric molecule, aids in the incorporation of therapeutic agents for their delivery^33,34^. In addition to this, the use of stabilizer either ionic or non-ionic along with the PLGA is essential to attain physical stability of SGNCs formulation, with the mechanism of action of electrostatic repulsion and steric stabilization^35,36^. The nonionic surfactants and polymers that are commonly used as a stabilizer during the SGNCs formulation are Poloxamer 188, Tween 80, polyethylene glycol, polyvinylpyrrolidone and cellulose derivatives such as hydroxypropyl cellulose and hydroxypropyl methylcellulose. As per the literature review, the major role of stabilizer in SGNCs formulation is to wet the drug particles, preventing Ostwald ripening and agglomeration of the nanocrystal, to a physically stable formulation by the formation of steric or an ionic barrier^37–39^. Typically, the bottom-up and top-down techniques, as well as the combinations of both can produce SGNCs. Second generation smarter nanocrystals of oral hypoglycemic, BCS class II drugs are achieved by using biodegradable polymers, stabilizer and drug, employing specialized pre-combinative treatment strategies such as Emulsion diffusion - high-pressure homogenization - Solvent evaporation (EHS) techniques^40–42^.

This research study postulated that gliclazide, oral hypoglycemic BCS class II drug loaded PLGA Second generation smarter nanocrystals may overcome the drug delivery challenges such as poor solubility, low and variable bioavailability, irreproducible therapeutic response, a large amount of oral dose for conventional dosage. Therefore, the present study is aimed to design, develop and delivery of gliclazide SGNCs, produced by pre-combinative strategies such as emulsion diffusion, high-pressure homogenization and solvent evaporation method using PLGA biodegradable polymers in combination with a stabilizer such as Poloxamer 188, PEG 4000, HPMC-E15. Taguchi experimental design was employed in design and fabrication of gliclazide SGNCs using Gliclazide -PLGA ratio at 1:0.5, 1:0.75, 1:1 with stabilizer such as Poloxamer 188, PEG 4000, HPMC (0.5, 0.75, 1% w/v). The formulated gliclazide SGNCs were investigated on physicochemical properties, *in vitro* drug release and *in vivo* performance such as pharmacokinetic-pharmacodynamic correlation, therapeutic efficacy and bioavailability studies using streptozotocin/nicotinamide-induced type-2 diabetes rat model^43,44^.

## Results and Discussion

The present therapy management of chronic T2DM is predominantly associated with frequent administration of oral hypoglycemic agents. Multiple dosing, side effects and poor patient compliance of oral hypoglycemic agents are the major challenges in successful design, delivery and therapy management of oral hypoglycemic agents. Gliclazide, a clinically safe and approved first line of drug for T2DM management was selected as a model drug for the study. Conventional gliclazide tablets produce poor and erratic bioavailability, irreproducible therapeutic response, need frequent administration of a large amount of oral dose in T2DM, due to its low solubility in the stomach and variable absorption in the intestine. A conventional gliclazide oral dosage form also failed to meet the typical physiological goal of T2DM, such as basic needs between meals and during the night, which demands a faster drug release followed by prolonged drug release profile to maintain constant plasma glucose level over an extended period. To overcome formulation and drug delivery challenges, poor bioavailability, poor glycemic control and poor patient compliance associated with gliclazide, oral hypoglycemic BCS class II drug, second-generation smarter functional polymeric nanocrystals novel delivery system was adopted. The main objective of this research study was to explore the advanced concept and technology of second-generation smarter functional polymeric nanocrystal in the design, development, and delivery of gliclazide SGNCs in T2DM management.

### Formulation and Optimization of Gliclazide loaded PLGA SGNCs

Gliclazide SGNCs were produced by pre-combinative (bottom-up and top-down technology) treatment strategies such as single emulsion diffusion-high pressure homogenization-solvent evaporation. In Taguchi experimental design, gliclazide -PLGA ratio, type of stabilizer, percentage stabilizer and high-pressure homogenization cycles are considered as formulation independent variables in the design and development of gliclazide SGNCs formulations. Gliclazide SGNCs were formulated using gliclazide-PLGA ratio at 1:0.5, 1:0.75, 1:1 with stabilizer such as Poloxamer 188, PEG 4000, HPMC E15 (0.5, 0.75, 1% w/v). As per the experimental design, 9 different formulations of gliclazide SGNCs were developed and their physicochemical characterizations such as particle size, polydispersity index, zeta potential, and entrapment efficiency were evaluated, shown in Table 1.

**Table 1:** Different batches of gliclazide loaded PLGA SGNCs formulations and their physicochemical characterizations.

Formulation code	PS ± SD (nm)	PDI ± SD	ZP ± SD (mV)	EE(%) ± SD
SGNCF1	106.3 ± 2.69^c,f,h^	0.222 ± 0.104	−18.2 ± 1.30^b,c,e,f,i^	86.27 ± 0.222
SGNCF2	114.7 ± 2.77^c,f,h^	0.216 ± 0.004	−23.1 ± 2.08^a,c,d,e,f,g,h,i^	84.40 ± 0.222
SGNCF3	220.2 ± 8.50^a,b,d,e,g,h^	0.284 ± 0.022	−8.80 ± 0.66^a,b,d,e,g,h^	91.50 ± 0.007
SGNCF4	120.0 ± 4.11^c,f,h^	0.221 ± 0.104	−17.2 ± 1.57^b,c,e,f,i^	84.13 ± 0.001
SGNCF5	94.89 ± 5.36^c,f,h^	0.217 ± 0.010	−18.4 ± 1.15^b,c,e,f,i^	85.87 ± 0.002
SGNCF6	176.8 ± 6.92^a,b,c,d,e,g,h,i^	0.218 ± 0.007	−11.6 ± 1.00^a,b,d,e,g,h^	79.65 ± 0.006
SGNCF7	102.5 ± 0.53^c,f,h^	0.228 ± 0.002	−18.3 ± 1.15^b,c,e,f,i^	84.10 ± 0.003
SGNCF8	110.1 ± 9.01^c,f,h^	0.161 ± 0.010	−18.4 ± 0.44^b,c,e,f,i^	87.40 ± 0.001
SGNCF9	223.4 ± 3.03^a,b,d,e,f,g,h,i^	0.245 ± 0.004	**−**10.4 ± 1.04^a,b,d,e,g,h^	79.03 ± 0.010

PS: particle size; PDI: polydispersity index; ZP: zetapotential; EE: entrapment efficiency; SD: standard deviation; (n = 3). Samples were analysed using ANOVA Dunnett’s C statistical test. ^a^P < 0.05 vs. SGNCF1; ^b^P < 0.05 vs. SGNCF2; ^c^P < 0.05 vs. SGNCF3; ^d^P < 0.05 vs. SGNCF4; ^e^P < 0.05 vs. SGNCF5; ^f^P < 0.05 vs. SGNCF6; ^g^P < 0.05 vs. SGNCF7; ^h^P < 0.05 vs. SGNCF8; ^i^P < 0.05 vs. SGNCF9.

All nine formulations of gliclazide loaded PLGA SGNCs were successfully manufactured by employing pre-combinative treatment strategies that could produce nanoparticle of mean particle size (DLS) in the range of 94.89 ± 5.36 nm to 223.4 ± 3.03 nm. All the formulations of gliclazide loaded PLGA SGNCs exhibited negative zeta potential, from −8.80 ± 0.66 mV to −23.1 ± 2.08 mV and polydispersity index range from 0.161 ± 0.010 to 0.284 ± 0.022, indicates good stability and ideal homogenous distribution pattern. Gliclazide entrapment efficiency of all nine formulations SGNCs were found in the range of 79.03 ± 0.010% to 91.50 ± 0.007%. To further optimize SGNCs formulation, the particle size and zeta potential data were further analyzed to ANOVA Dunnett’s C, statistical test, p < 0.05 study. The study reveals that the particle size of formulation SGNCF1 (Poloxamer 188, used as a stabilizer) is significantly different to other formulations where HPMC E15, is used as a stabilizer (SGNCF3, SGNCF6, SGNCF9) (ANOVA Dunnett’s C statistical test, p < 0.05). Similarly, in case of zeta potential, the formulations with least significant difference (ANOVA Dunnett’s C statistical test, p < 0.05) are SGNCF1, SGNCF4, SGNCF5, SGNCF7 and SGNCF8, able to provide satisfactory short-term stabilization of SGNCs, without any significant aggregation or agglomeration of the nanoparticles.

**Figure 1:**
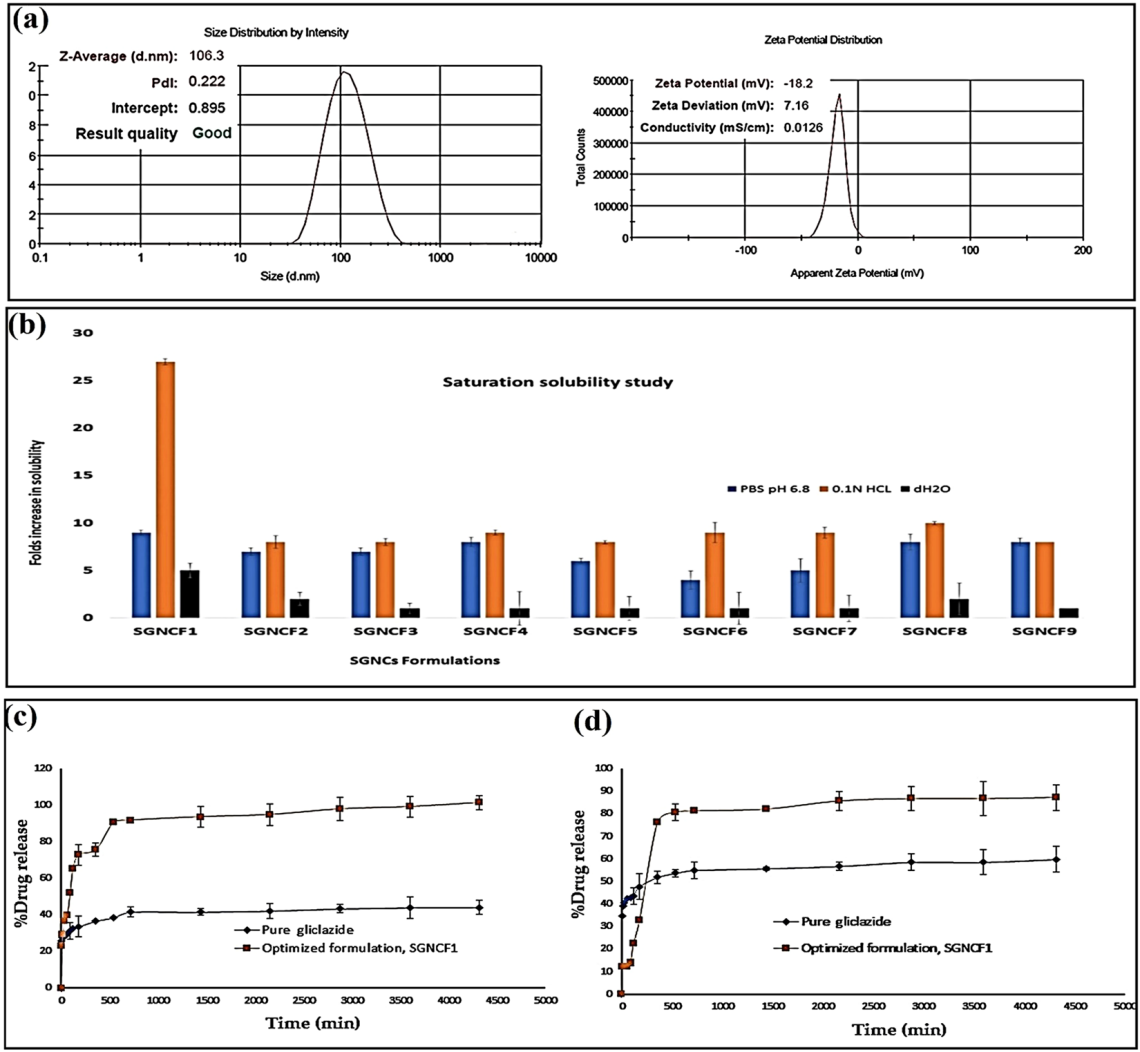
**(a)** Particle size distribution profile and zeta potential distribution profile of optimized SGNCF1 by photon correlation spectroscopy; (**b**) Represents saturation solubility study of gliclazide loaded PLGA SGNCs formulations in different mediums, phosphate buffer pH 6.8., 0.1 N hydrochloric acid pH1.2 and distilled water; (**c**) Represents dissolution profiles of pure gliclazide and optimized formulation, SGNCF1, at 0.1 N hydrochloric acid, pH 1.2 and **(d)** phosphate buffer, pH 6.8.

The variation in mean particle size, zeta potential, entrapment efficiency and their reproducibility of gliclazide loaded PLGA SGNCs formulations, signifies that these responses are strongly dependent on the independent variables that are selected within the space of Taguchi orthogonal experimental design for SGNCs formulations. Considering the potential of small particle size, good zeta potential, ideal polydispersity index with high entrapment efficiency, the formulation SGNCF1, developed with gliclazide: PLGA 1: 0.5 ratio with 0.5% w/v Poloxamer-188, as a stabilizer, produced optimized SGNCs formulation. SGNCF1 formulation showed small mean particle size (106.3 ± 2.69 nm), good zeta potential (−18.2 ± 1.30 mV), small PDI (0.222 ± 0.104) (see Fig. 1a) and high entrapment efficiency (86.27 ± 0.222%). SGNCF1 has shown highest entrapment efficiency, indicates gliclazide was entrapped into PLGA and emulsified by aqueous Poloxamer 188 yields a relatively high entrapment efficiency possibly due to the strong interaction of gliclazide and polymer PLGA. HPMC E15, used as a stabilizer (SGNCF3, SGNCF6, SGNCF9) with PLGA SGNCs formulations produced larger particle size with poor zeta potential compared to other stabilizers Poloxamer-188 and PEG 4000, evident that nature and concentration of stabilizer play a significant role in particle size and stability of SGNCs formulations. SGNCF1 the optimized formulation with small particle size, good zeta potential, low polydispersity index, high entrapment efficiency, expected to improve the solubility, bioavailability and therapeutic efficacy of gliclazide, which was evaluated for further confirmation^45^.

### Solubility of gliclazide loaded PLGA SGNCs

The solubility of the pure gliclazide and all nine formulations of gliclazide loaded PLGA SGNCs were carried out comparatively in three different mediums, in phosphate buffer pH 6.8, 0.1 N hydrochloric acid pH 1.2 and distilled water, shown in Table 2. The saturation solubility of pure drug gliclazide in distilled water, 0.1 hydrochloric acid (pH 1.2) and phosphate buffer pH 6.8 were found as 35.87 µg/ml, 107.80 µg/ml and 411.957 µg/ml respectively. It is evident that pure gliclazide was poorly soluble in water. The highest solubility of pure gliclazide was observed in phosphate buffer, whereas for optimized SGNCs formulation, SGNCF1 shown a significant increase in the solubility (27 folds) in 0.1 N hydrochloric acid. The rest of the SGNCs formulations analogously produced higher fold of solubility in 0.1 N hydrochloric acid (pH 1.2) followed with phosphate buffer (pH 6.8) and distilled water, depicted in Fig. 1b. This hypothesized that pure gliclazide is a hydrophobic, weak acid with a lower solubility in acidic medium, hence enhancing solubility in acidic medium is crucial for the improvement of dissolution rate as well as the bioavailability of the drug. However, after formulating gliclazide loaded with PLGA, a polymer composed of equal ratios of glycolic acid and lactic acid in combination with second-generation nanocrystal approach of nanonization, gliclazide release accelerated in acidic environment, maybe due to synergic effects of homogenously distributed PLGA SGNCs with Poloxamer 188 as stabilizer, has membrane permeability enhancing effect with bipolar amphipathic dispersible effects that promotes the solubility of gliclazide^46,47^.

**Table 2:** Folds increase in solubility and percentage drug release for different gliclazide loaded PLGA SGNCs formulations at different media.

Gliclazide/Formulation code	Folds increase in solubility ± SD			Percentage drug release (%)			
	phosphate buffer pH 6.8	0.1 N hydrochloric acid pH 1.2	Distilled water	0.1 N hydrochloric acid pH 1.2		phosphate buffer pH 6.8	
				2 h	72 h	2 h	72 h
Gliclazide	—	—	—	32.416	44.0367	43.592	59.871
SGNCF1	9 ± 0.268	27 ± 0.3257	5 ± 0.7787	65.443	101.3147	22.403	87.261
SGNCF2	7 ± 0.3678	8 ± 0.657	2 ± 0.677	45.872	70.336	23.953	80.801
SGNCF3	7 ± 0.36787	8 ± 0.378	1 ± 0.553	21.938	48.971	18.214	60.162
SGNCF4	8 ± 0.49678	9 ± 0.268	1 ± 1.788	32.110	65.138	23.380	78.902
SGNCF5	6 ± 0.25767	8 ± 0.167	1 ± 1.267	24.253	60.612	27.515	71.906
SGNCF6	4 ± 0.956	9 ± 1.077	1 ± 1.687	22.070	53.078	26.767	65.463
SGNCF7	5 ± 1.2378	9 ± 0.578	1 ± 1.378	22.974	55.525	26.731	67.051
SGNCF8	8 ± 0.847	10 ± 0.1678	2 ± 1.678	51.988	76.453	21.370	84.160
SGNCF9	8 ± 0.4213	8 ± 0.2767	1 ± 1.906	21.733	50.239	25.966	60.615

SD: standard deviation; (n = 3).

### Shape and morphology characterization of gliclazide loaded PLGA SGNCs

**Figure 2:**
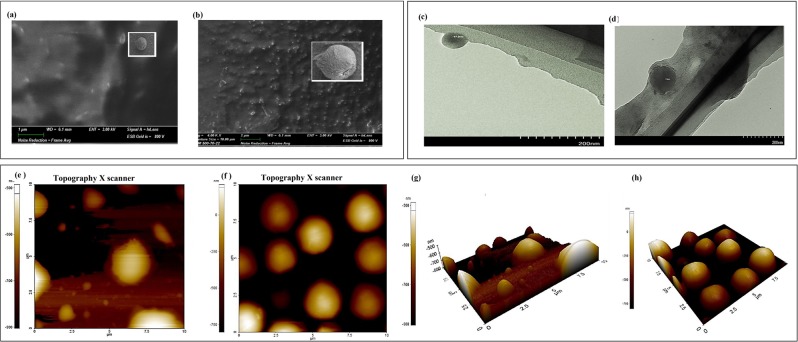
Shape and morphology characterization by field emission scanning electron microscopy (FESEM) images of **(a)** placebo of SGNCF1 and **(b)** optimized formulation, SGNCF1; Transmission electronic microphotographs (TEM) of **(c)** placebo of SGNCF1 and **(d)** optimized formulation, SGNCF1; Atomic force microscopy (AFM) images of **(e)** 2D view of placebo of SGNCF1 and **(f)** 2D view of optimized formulation, SGNCF1; **(g)** 3D view of placebo of SGNCF1 and **(h)** 3D view of optimized formulation, SGNCF1.

The shape and morphology of gliclazide loaded PLGA SGNCs optimized formulation SGNCF1 and its placebo was characterized using field emission scanning electron microscope (FESEM), transmission electron microscopy (TEM) and atomic force microscopy (AFM) (see Fig. 2). FESEM images (see Fig. 2a,b) of particles of SGNCs optimized formulation and placebo, show homogenous and uniformly dispersed, rough porous surface, almost spherical shape with a low tendency for aggregation of PLGA SGNCs. The mild aggregation of the SGNCs may be due to the operational procedure of freeze-drying. The average diameter of the optimized gliclazide loaded PLGA SGNCs formulation, SGNCF1 ranged from 90 to 110 nm, whilst the placebo was ranging from 50 to 70 nm. The FESEM study, also indicates that placebo PLGA SGNCs produced smaller particle size is compared to gliclazide loaded PLGA SGNCs, same findings also affirmed by TEM study. The transmission electronic microphotographs (TEM) of PLGA SGNCs, showed dark staining enclosed with bright lining indicate the presence of spherical polymeric particle of PLGA nanocrystal, as depicted in Fig. 2c,d. TEM image of optimized formulation, SGNCF1 shows a little larger particle size (114 nm) compared to particle size obtained from PCS, zetasizer (around 106.3 nm), which can be explained by aggregation of the nanoparticles due to the high freezing rate causes the bonds to be broken resulting on aggregation and enlargement of particle size during the freeze-drying process. The placebo TEM microphotograph shows smaller particle size (67.2 nm) compared to gliclazide loaded PLGA SGNCs optimized SGNCF1 formulation^48^.

The size, morphology and distribution pattern of nanoparticles of optimized formulation SGNCF1 and its placebo were further visualized by two dimensional (2D) and 3 dimensional (3D) images by atomic force microscopy (AFM) (see Fig. 2e–h). The particle size of optimized formulation SGNCF1 and placebo were determined using AFM, which illustrates the average size distribution of 10 to 13 nm for the optimized formulation, whereas 2 to 14 nm for placebo. Particle size determined by AFM represents the size of the core of the nanoparticle of PLGA SGNCs which is much smaller in contrast to particle size reported by PCS, zetasizer, characterizes hydrodynamic diameter of PLGA nanoparticle^49^. The AFM image analysis further emphasizes that PLGA SGNCs placebo shown broader, irregular particle size distribution with spherical morphology, whereas optimized formulation SGNCF1 has narrow particle size with homogenous distribution and spherical shaped surface morphology.

### X-ray diffraction (XRD), differential scanning calorimetric (DSC) and fourier transform infrared (FTIR) spectroscopy studies

**Figure 3:**
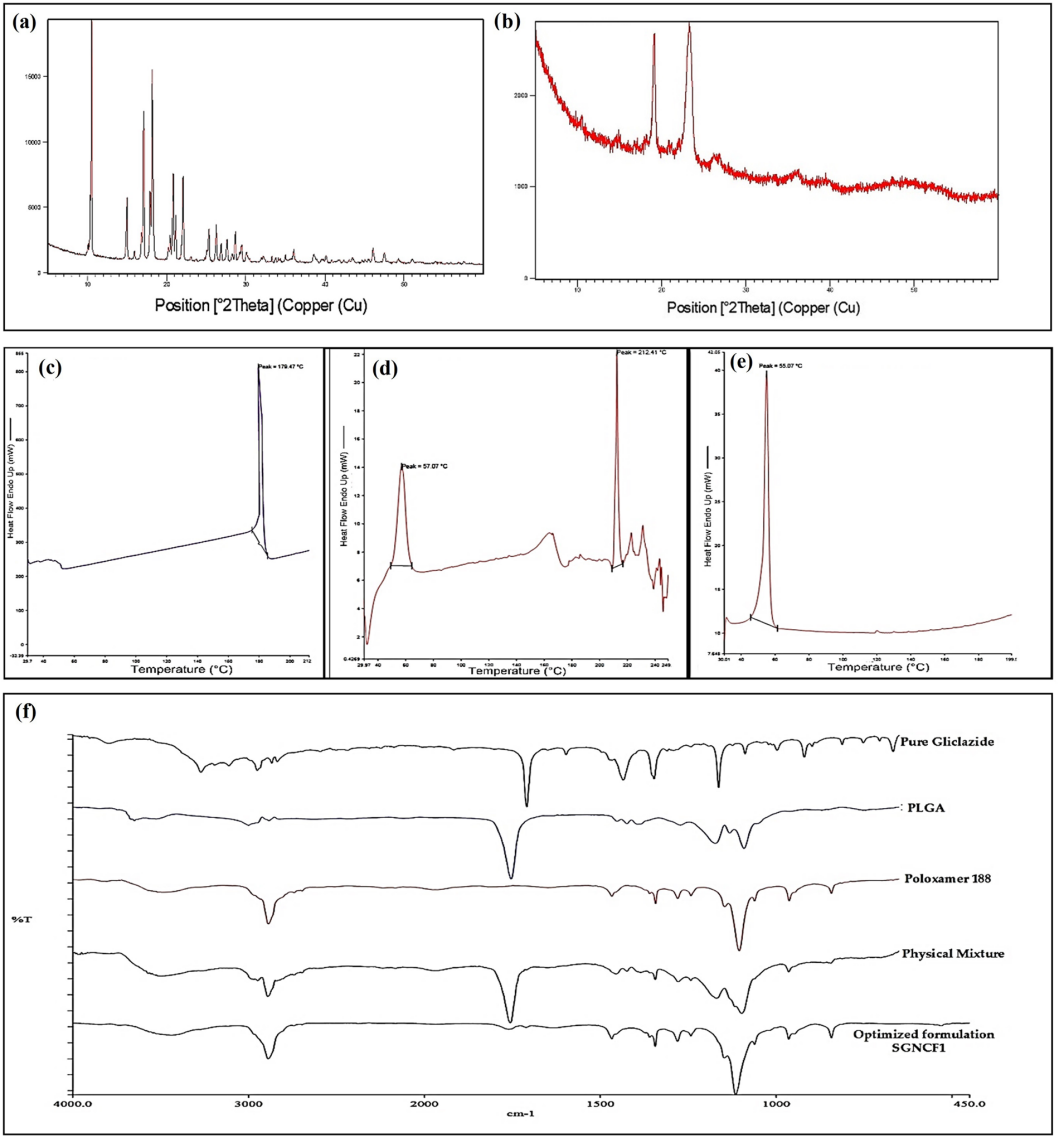
X-ray diffractograms of **(a)** pure gliclazide, **(b)** optimized formulation, SGNCF1; DSC thermograms of **(c)** pure gliclazide, **(d)** physical mixture and **(e)** optimized formulation, SGNCF1; **(f)** FTIR spectra of pure gliclazide, PLGA, Poloxamer 188, physical mixture and optimized formulation, SGNCF1.

The X-ray powder diffractometry (XRD) analysis was performed for pure gliclazide and gliclazide loaded PLGA SGNCs optimized formulation, SGNCF1 to establish the impact of nanonization on crystallinity and phase changes in the internal structure of gliclazide loaded PLGA SGNCs, presented in Fig. 3a,b. XRD diffractogram pattern of pure gliclazide showed distinct characteristic peaks with significant intensity at 2θ of 10.51° to 14.99°, 17.08° to 20.43°, 20.8° to 23.71°, 36.06° to 40.12° and other several indistinct peaks with reduced intensity represents the crystallinity of pure gliclazide. In contrast, the analysis of XRD diffractogram of lyophilized gliclazide loaded PLGA SGNCs optimized formulation, SGNCF1 showed less and reduced distinct characteristic peaks with significant intensity at 2θ of 19.0975°, 23.3997°, 36.0467°, 39.7044°, indicate gliclazide still partially retained its crystallinity in PLGA SGNCs formulation. The presence and absence of new distinct peaks with high intensity of optimized formulation SGNCF1 diffractogram signify that the drug gliclazide has been successfully loaded in PLGA SGNCs formulation stabilized with Poloxamer 188. The partial changes in the molecular conformation of gliclazide from crystallinity to amorphous polymorphs create a favorable environment for enhancing solubility, dissolution rate and bioavailability of gliclazide, a BCS II drug^50^.

To investigate the physical state of drug and excipients compatibility in the formulation development of SGNCs, a comparative DSC thermal analysis (Fig. 3c–e) was performed for pure gliclazide, physical mixer and gliclazide loaded PLGA SGNCs optimized formulation, SGNCF1. Pure gliclazide thermogram exhibited a sharp characteristic endothermic peak at 179.47 °C, which corresponds to a single transition temperature (melting point) of gliclazide. Whereas the DSC thermogram of the physical mixture of gliclazide, PLGA and poloxamer 188, showed a minor shift of drug peak to 212.41 °C, this might be due to the possible physical interaction of gliclazide crystals with excipients used in nanocrystal formulation. Thermogram scan of gliclazide loaded PLGA SGNCs optimized formulation SGNCF1, produced a single sharp endothermic peak at 55.07 °C indicate a sharp change in transition temperature and reduction for gliclazide crystallinity in PLGA SGNCs formulation. The DSC analysis also emphasizes that gliclazide forms a thermodynamically stable system by making strong interaction and molecular dispersion in PLGA and poloxamer 188^51^. This DSC study finding falls in line with the results of X-ray diffractogram analysis of PLGA SGNCs formulation, which confirmed the hypothesis of gliclazide retained as a nanocrystal form with molecular dispersion in PLGA polymer.

Fourier transform infrared spectroscopic analysis was conducted for pure gliclazide, PLGA, Poloxamer 188, physical mixture and optimized formulation, SGNCF1. The FTIR spectrum of pure gliclazide exhibited characteristic peaks at 1164.06 cm^−1^ (Sulphonyl S=O stretching),1354.05 cm^−1^ (SO2NH stretching), 1597.09 cm^−1^ (Secondary amine N-H bending), 1709.92 cm^−1^ (Acyclic ketone carbonyl (C=O) stretching), 3113.16 cm^−1^ (=CH stretching) and 3275.19 cm^−1^ (Secondary amine N-H stretching) respectively. Spectral comparative analysis of physical mixture, optimized formulation, SGNCF1, PLGA, Poloxamer 188 for pure gliclazide spectrum shows a complete absence of pure gliclazide characteristic peaks in the spectrum of optimized formulation, SGNCF1, represented in Fig. 3f. This absence of characteristic peaks of gliclazide in SGNCs formulation confirms that the drug gliclazide was encapsulated into the core of Poloxamer coated PLGA polymeric nanocrystals. This FTIR studies also established, that there was no potential interaction between drug and other excipients used in the development of SGNCs^52^.

### *In vitro* drug release studies

Drug release profile analysis of all SGNCs formulations along with pure gliclazide was performed and reported in Table 2. Comparative dissolution studies of pure drug with an optimized formulation in acidic and alkaline media were depicted in Fig. 1c,d. The optimized formulation, SGNCF1 has shown significant enhancement in dissolution rate in both dissolution media at pH 1.2 and pH 6.8, at two different time points, 2 h and 72 h compared to pure gliclazide and other SGNCs formulations. The nanonized polymeric nanocrystal with good zeta potential and low polydispersity index of the optimized formulation SGNCF1 enhances solubility and dissolution rate of poorly soluble drug gliclazide, this could be attributed to the increased surface area by nanonization of SGNCs. Percentage of drug release for SGNCF1 at 2 h and 72 h in acidic medium, 0.1 N hydrochloric acid pH 1.2 was 65.43%, 101.31% respectively. Whereas, in phosphate buffer, pH 6.8 medium, the percentage of drug release for SGNCF1 was 22.40% at 2 h and 87.26% at 72 h respectively. It was also evident from the dissolution study of optimized formulation SGNCF1, that more than 70% drug was released during the first 3 h from the acidic medium, compared to the alkaline medium. This difference in drug release rate in different acidic and alkaline media is due to rapid degradation of PLGA in acidic medium, where the equivalent ratio of lactic acid and glycolic acid were auto catalyzed over a period of time and increase in both concentrations may result in initial quick release followed by constant delayed release of drug gliclazide. Furthermore, dissolution profiles of the optimized formulation, SGNCF1 in both the acidic and alkaline medium exhibit a biphasic drug release of initial immediate release followed by delayed-release, may be contributed to the instant dissolution of surface deposited gliclazide on PLGA SGNCs followed by delayed drug release from the encapsulated core of polymeric nanocrystal^53^. The optimized gliclazide loaded PLGA SGNCF1 formulation demonstrates that the *in vitro* drug release performance was greatly attributed by particle size, dissolution rate, nature and proportion of polymer and stabilizer used in SGNCs formulation.

### *In vivo* performance study

**Figure 4:**
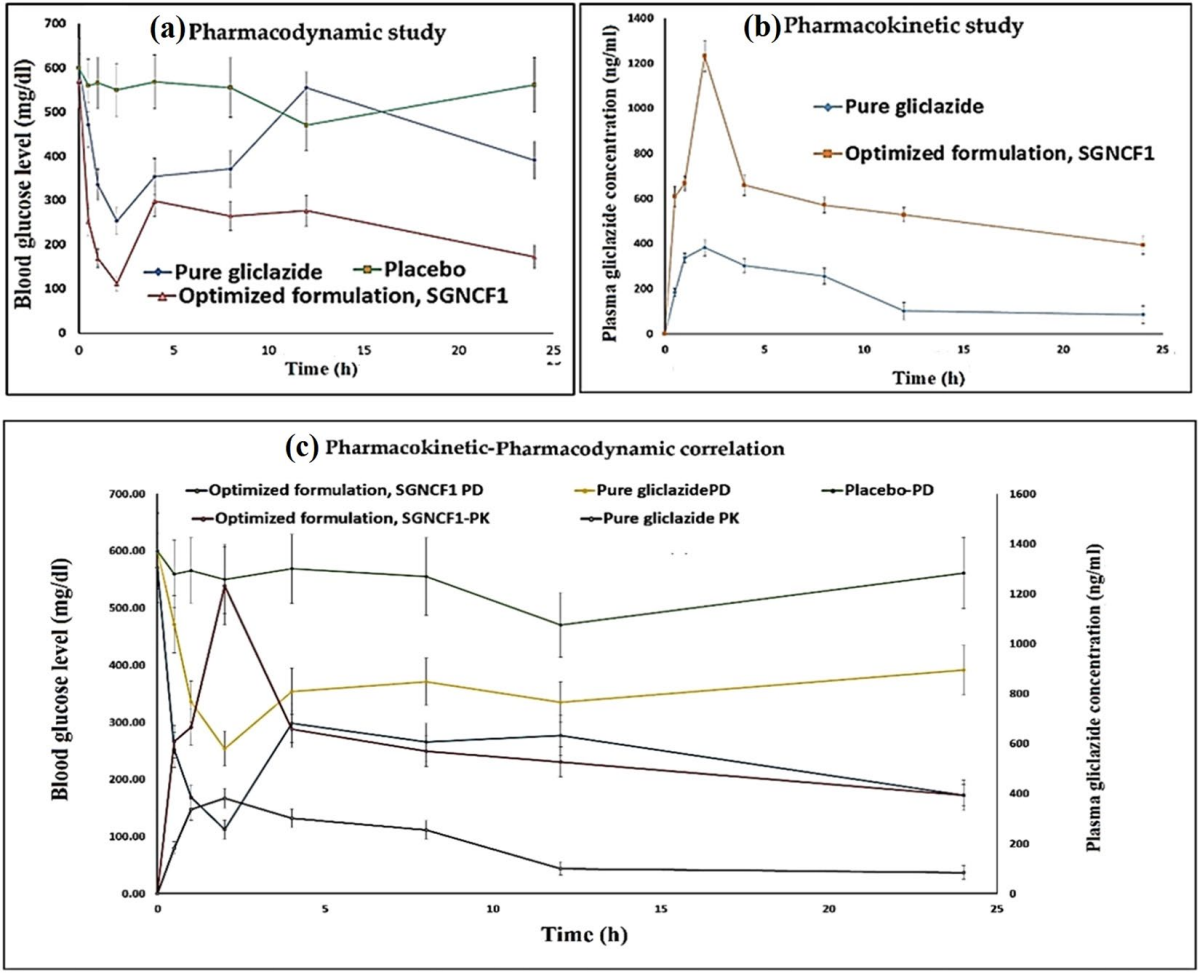
Represents the *in vivo* performance study of gliclazide loaded PLGA SGNCs formulation, (**a**) Pharmacodynamic responses of pure gliclazide, placebo and optimized formulation, SGNCF1 on type II diabetic rats; (**b**) Plasma mean gliclazide concentration–time curves after single oral dosing of pure gliclazide and optimized formulation, SGNCF1 on type 2diabetic rats; (**c**) Represents Pharmacokinetic-Pharmacodynamic correlation plot of gliclazide loaded PLGA SGNCs formulation on type 2 diabetic rats. Each point represents the mean ± SD of the data obtained from the experiments (n = 6).

The *in vivo* performance concerning pharmacodynamics, pharmacokinetics, bioavailability and therapeutic efficacy of developed gliclazide loaded PLGA SGNCs were investigated on STZ-NA induced type-2 diabetes rat model. The established, HPLC bioanalytical method with regression coefficient (r2) value 0.9963 and gliclazide retention time 5.45 min was adopted in the estimation of plasma gliclazide concentration used in pharmacokinetics and bioavailability study. The impact of second-generation nanocrystal formulation on gliclazide *in vivo* performance was established with the type-2 diabetes rat model as depicted in Fig. 4a,b. The pharmacodynamic study in type-2 induced diabetes rat model of pure gliclazide and optimized formulation, SGNCF1 reveals that pure gliclazide reduced blood glucose level from 600 ± 67.1 mg/dl to 254 ± 30.4 at 2 h and from 600 ± 67.1 mg/dl to 391.3 ± 42.8 by the end of 24 h, whereas in compared to pure gliclazide, the optimized formulation, SGNCF1 significantly reduced the blood glucose level from 570.7 ± 60.7 mg/dl to 112.3 ± 15.9 at 2 h and from 570.7 ± 60.7 mg/dl to 172.7 ± 26.5 by 24 h respectively. This initial quick decrease following a sustained way of reduction in blood glucose level by optimized formulation, SGNCF1 would be contributed to the initial fast dissolution and absorption of drug gliclazide from second generation nanocrystal formulation followed by delayed drug release from the polymeric system of PLGA stabilized with poloxamer 188. This pattern of reduction in blood glucose was further evidenced and confirmed from *in vitro* dissolution profile of optimized formulation, SGNCF1^54^.

The average mean of plasma gliclazide concentration versus time was analysed for pharmacokinetic and bioavailability study of the pure drug (API) and optimized nanoformulation, SGNCF1, in type II diabetic induced rats. The plasma gliclazide concentration versus time data was exposed further to the non-compartment model in the determination of model-independent pharmacokinetic parameters, presented in Table 3. The plasma gliclazide concentration versus time profile of optimized nanoformulation SGNCF1 reflects that gliclazide plasma concentration reached to maximum by 2 h with quick absorption of the drug, followed by delayed prolonged drug absorption from PLGA based nanocrystals in comparison to pure gliclazide concentration in plasma. The faster absorption of gliclazide from SGNCs was proved with parameter Tmax (h) (2 ± 1 h). The optimized SGNCF1 formulation produced significant increase in Cmax (1232.19 ± 124.3949 µg/ml), AUC total (30.291 ± 1.426186 µg/ml × h), AUMC total (1257.52 ± 128.5785 µg/ml × (h)^2^) compared to pure gliclazide, Cmax (382.182 ± 15.14 µg/ml), AUC total (5.3485 ± 0.51 µg/ml × h), AUMC total (80.32 ± 5.10 µg/ml × (h)^2^). The optimized SGNCF1 formulation has shown 5.71 times increase in AUC total and 15.71 times in AUMC total compared to pure gliclazide, reflects the significant improvement in bioavailability by second generation nanocrystal approach of nanonization of gliclazide. The elimination half-life (t- half) and MRT (h) of optimized formulation were observed as 29.56 h, 41.51 h, which is approximately 3 times longer than the half-life (t-half, 9.75 h) and MRT (15.019 h) of pure gliclazide. The elimination rate constant (β) of optimized formulation SGNCF1 and pure gliclazide were observed as 0.071 h^−1^, 0.023 h^−1^ respectively. This prolongation of the residence time of the drug in the body, from optimized SGNCs formulation, indicates the delayed steady state delivery of gliclazide from PLGA nanocrystal for better therapeutic efficacy in T2DM management. Additionally, the pharmacokinetic-pharmacodynamic correlation of pure gliclazide, optimized formulation, SGNCF1and placebo showed a better agreement and good point to point correlation of plasma gliclazide concentration with a reduction in plasma glucose in a type-2 diabetes induced rat model was established (see Fig. 4c)^55^.

**Table 3:** Summary of PK parameters of gliclazide and optimized formulation, SGNCF1 on type II diabetic rats after single oral dose administration.

PK parameters	Pure gliclazide	SGNCF1
Cmax (ng/ml)	382.182 ± 15.140	1232.19 ± 124.39
Tmax (h)	2 ± 1	2 ± 1
t half (h)	9.75 ± 0.55	29.56 ± 3.53
Cl (ml/h)	1503.87 ± 0.036	265.539 ± 0.005
Vss (ml)	22586.6 ± 0.12	11023.7 ± 0.10
AUC total (µg/ml × h)	5.3485 ± 0.51	30.291 ± 1.42
AUMC total (µg/ml) × (h)^2^	80.32 ± 5.104	1257.52 ± 128.578
MRT (h)	15.019 ± 2.740	41.51 ± 1.593
β (1/h)	−0.071 ± 0.003	−0.023 ± 0.0069

SD: standard deviation; (n = 6).

Physical stability of optimized SGNCF1 formulation was performed by measuring particle size, zeta potential and polydispersity index using short term stability studies at 4 °C, 25 °C and 40 °C for 30 days. The stability study revealed that the lyophilized gliclazide loaded PLGA SGNCs showed no significant changes in the particle size, zeta potential, and polydispersity index of optimized SGNCF1 formulation in a short-term stability study for 30 days. This study emphasizes that SGNCs formulation successfully designed and developed a stable second generation nanocrystal formulation in the fabrication of PLGA with Poloxamer 188 as a stabilizer in drug delivery of gliclazide. The overall findings of the study highlight the significance of second-generation nanocrystals and its potential implication *in vitro* and *in vivo* performance of gliclazide to enhance the solubility, absorption, poor and erratic bioavailability and therapeutic efficacy with a low oral dose of gliclazide, BCS II, first-line drug in T2DM.

## Conclusions

The prime objective of this study was to design, develop and explore the potential application of second-generation polymeric nanocrystal technology to overcome the formulation and drug delivery challenges associated with gliclazide, a first-line oral hypoglycemic, BCS class II drug used in T2DM management. A stable, gliclazide loaded PLGA second generation smarter nanocrystal carrier stabilized with poloxamer 188 was successfully formulated by employing specialized pre-combinative nanoformulation strategies. The optimized gliclazide loaded PLGA second-generation formulation SGNCF1 was developed by employing Taguchi experimental design and characterized for particle size, zeta potential, solubility studies, drug entrapment, surface morphology studies, *in vitro* drug release studies, drug excipient compatibility study, *in vivo* performance study and stability studies. The results of *in vitro* and *in vivo* study demonstrated that the optimized gliclazide loaded PLGA second-generation formulation SGNCF1, improved solubility, dissolution rate and bioavailability of gliclazide. PLGA second generation smarter nanocrystals exhibit a unique biphasic pattern of drug release that is an initial immediate drug release followed by delayed-release, which was further confirmed with *in-vitro* drug release and PK-PD correlation study. This unique pattern of drug release of PLGA second generation smarter nanocrystals can be explored to meet the typical physiological needs of T2DM patients, such as a faster drug release at the time of meals followed by prolonged drug release profile over an extended period of time to maintain constant plasma glucose level, is highly desirable for better patient compliance and drug therapy management. The overall findings of the study envisioned that second-generation PLGA based nanocrystal holds greater potential for gliclazide drug delivery to T2DM management in particular and which can be extended for other BCS II categories of therapeutic agents delivery in general.

## Materials and Methods

### Materials

Gliclazide was purchased from Toronto Research Chemicals Inc, Canada. Streptozotocin supplied by Biosyntech Group Sdn. Bhd., Malaysia. Nicotinamide was obtained from Santa Cruz Biotechnology, USA. Poly(lactic-co-glycolide), L/G:50:50, ester terminated, MW 7000–17000), polyethylene glycol 4000 (PEG 4000), hydroxypropyl methylcellulose (HPMC E 15) was purchased from Sigma Aldrich, St Louis, MO, USA. Poloxamer 188 (Ph. Eur., NF grade) and acetone (EMSURE ACS, ISO, Reag. Ph. Eur) were obtained from Merck Sdn Bhd, Selangor, Malaysia. All other chemicals and reagents used were of analytical grade.

### Taguchi orthogonal array design for gliclazide loaded PLGA SGNCs

Taguchi design of the experiment was adopted in the formulation, optimization and evaluation of the influence of independent variables on the development of gliclazide loaded PLGA SGNCs. Drug: PLGA polymer ratio, stabilizer type, percentage of stabilizer and homogenization cycles were selected as independent variables, which on the ground of their paramount influence on particle size distribution, drug entrapment efficiency and drug release, were considered as dependent variables of SGNCs. To minimize the number of experiments, Taguchi orthogonal array table was designed by employing these four independent variables with three different levels in L type orthogonal array, which could affect the dependent variables of SGNCs. Independent variables and their levels employed in Taguchi orthogonal array design used in the formulation of SGNCs as shown in Table 4. In brief, Taguchi orthogonal array design (4 parameters and 3 levels) indicates that 9 experiments were required to study the effect of all parameters involved in achieving the desired target output such as particle size, entrapment efficiency, and drug release, were considered as dependent variables of gliclazide loaded PLGA SGNCs formulation. Table 5 represents the compositions of all different coded 9 experimental runs of gliclazide loaded PLGA SGNCs formulations, developed according to Taguchi experimental design. All 9 experimental runs of SGNCs formulations were performed in triplicate^56–58^.

**Table 4:** Taguchi L type orthogonal array design for four independent variables at three different levels employed in the production of second generation nanocrystals.

Independent variable	Level 1	Level 2	Level 3
A- Gliclazide: PLGA	1:0.5	1:0.75	1:1
B-Stabilizer type	Poloxamer 188	PEG 4000	HPMC(E15)
C-Percentage stabilizer	0.5	0.75	1.0
D-Homogenization cycles at 1000 bar	5	10	20

PLGA: poly(D,L-lactide-co-glycolide); PEG 4000: Polyethylene glycol 4000; HPMC(E15): hydroxypropyl Methylcellulose.

**Table 5:** Different batches of formulations and their composition used in gliclazide loaded PLGA second-generation nanocrystals preparation using Taguchi L type orthogonal array design.

Formulation code	Gliclazide: PLGA ratio	Stabilizer type	Percentage of Stabilizer (%w/v)	Homogenization cycles at 1000 bar
SGNCF1	1:0.5	Poloxamer 188	0.5	5
SGNCF2	1:0.5	PEG 4000	0.75	10
SGNCF3	1:0.5	HPMC(E15)	1.0	20
SGNCF4	1:0.75	Poloxamer 188	0.75	20
SGNCF5	1:0.75	PEG 4000	1.0	5
SGNCF6	1:0.75	HPMC(E15)	0.5	10
SGNCF7	1:1	Poloxamer 188	1.0	10
SGNCF8	1:1	PEG 4000	0.5	20
SGNCF9	1:1	HPMC (E15)	0.75	5

PLGA: poly(D,L-lactide-co-glycolide); PEG 4000: Polyethylene glycol 4000; HPMC(E15): hydroxypropyl methylcellulose.

### Formulation and optimization of gliclazide loaded PLGA SGNCs

Taguchi experimental design was adopted in fabrication of SGNc using gliclazide -PLGA ratio at 1:0.5, 1:0.75, 1:1, stabilizer such as Poloxamer 188, PEG 4000, HPMC (0.5, 0.75, 1% w/v) with high pressure homogenization at 1000 bar (5,10,15 cycles). The gliclazide loaded PLGA organic phase was prepared by employing the single emulsion method. Briefly, accurately weighed 10 mg of gliclazide was added to the polymeric solution (PLGA in 2.4 ml of acetone) with gliclazide: PLGA in the ratio of 1:0.5, 1:0.75, 1:1, as per Taguchi experimental design. Gliclazide and PLGA were dissolved by the vortex mixer to form the organic phase. The aqueous phase was then formed by dissolving the stabilizer such as Poloxamer 188, PEG 4000, HPMC-E15 (0.5, 0.75, 1% w/v as per experimental design) in 20 ml of water. The organic phase was added into the aqueous phase under shear using high-speed homogenizer (IKA®T25 digital Ultra-Turrax®, Staufen, Germany) at 15000 rpm for 7 min to produce a coarse emulsion. Then the coarse emulsion was passed through a high-pressure homogenizer (Model M-110 P, Microfluidics, US) at 1000 bar (5, 10, 20 cycles) according to the Taguchi orthogonal experimental design. The nanosuspension was collected in a glass beaker and kept overnight on a magnetic stirrer (IKA-WERKE, RT10Power) at 300 rpm in a fume hood for solvent evaporation. The formulated nanosuspension of gliclazide loaded PLGA SGNCs was filtered using 0.20 µm filter (Minisart, Sartorius Stedim) and subsequently characterized for physicochemical evaluation such as particle size, zeta potential, polydispersity index, solubility, entrapment efficiency, freeze drying, surface morphology study, *in vitro* drug release and *in vivo* performance evaluation^31,59,60^.

### Nanoparticle size, size distribution and zeta potential measurements

Gliclazide loaded PLGA second-generation nanocrystals formulation was characterized for particle size (Z-average), polydispersity index and zeta potential with Malvern Zetasizer Nano series (Malvern Instruments, Germany). The particle size and polydispersity index were characterized by dynamic light scattering (DLS) principle, whereas zeta potential was estimated based on electrophoretic mobility under an electric field. Briefly, gliclazide loaded PLGA SGNCs were diluted with MilliQ water followed by vortex mix for 30 sec and analyzed. For each sample of gliclazide loaded PLGA SGNCs formulation, particle size, polydispersity index and zeta potential were run in triplicate to reduce random error^61^^.^

### Lyophilization of gliclazide loaded PLGA SGNCs

To assess the solubility, shape, and morphology of the gliclazide loaded PLGA SGNCs, suspension formulations were lyophilized. The gliclazide loaded PLGA SGNCs suspensions were first frozen at −80 °C for 2 days and then lyophilized using a freeze dryer (Labconco, Freezone 4.5, USA) at a controlled temperature of −50 °C and the pump operating at a vacuum pressure of 0.05 mbar over for 48 h. The lyophilized SGNCs samples were stored in an airtight container at room temperature for further analysis^62^.

### Saturation solubility study

The solubility of pure gliclazide and lyophilized gliclazide loaded PLGA SGNCs were determined by the shake-flask method. Excess amounts of gliclazide and lyophilized SGNCs were added with 2 ml of acetone and vortex mixed for 5 min. Then the samples were added into 50 ml separate centrifuge tubes containing 10 ml of solvent. The solvents screened for solubility studies were distilled water, 0.1 N hydrochloric acid at pH 1.2 and phosphate buffer at pH 6.8. The sealed tubes were then shaken in the orbital incubating shaker (WiseCube WIS-20, Daihan Scientific, South Korea) at 27 °C for 48 h to reach the equilibrium. The sample was then centrifuged (Eppendorf centrifuge 5424 R) at 10,000 rpm at 25 °C for 15 min and the resulting mixture was filtered through a membrane filter (0.45 µm, Sartorius) and the filtrate was suitably diluted accordingly and analyzed for their drug contents using UV spectrophotometer (UV–1900 spectrophotometer, Shimadzu, Tokyo, Japan) at 229 nm against the blank solutions. Each experiment was carried out in triplicate^62^.

### Entrapment efficiency

For the estimation of entrapment efficiency (% EE), 2 ml of sample SGNCs suspension was ultracentrifuged (Heraeus Multifuge X3R, Thermo Scientific, Reinach, Switzerland) at 15,000 rpm at 4 °C for 30 min. The clear supernatant was immediately analyzed for free drug content. The supernatant was diluted and analyzed by UV spectrophotometer (UV–1900 spectrophotometer, Hitachi, Tokyo, Japan) to determine the content of free drugs in the clear supernatant solution. The exact concentration of free drugs was determined from the standard curve of gliclazide. Each experiment was performed in triplicate^63^.

The % EE was calculated using the equation as below:

$$ \% \,{\rm{EE}}=\frac{{\rm{Initial}}\,{\rm{total}}\,{\rm{drug}}-{\rm{Free}}\,{\rm{drug}}}{{\rm{Initial}}\,{\rm{total}}\,{\rm{drug}}}$$

### Field emission scanning electron microscopy (FE-SEM)

The surface morphology of gliclazide loaded PLGA SGNCs formulation and its placebo PLGA SGNCs formulation were observed using field emission scanning electron microscope (Carl ZEISS GeminiSEM 500 Nano-twin lens). The lyophilized nanoparticles samples were sputter-coated with gold and dusted onto the double-sided tape on an aluminum stub at a current intensity of 40 mA for 30–40 s. Photomicrographs for both formulations were captured at the accelerated voltage of 1–5 kV^61^.

### Transmission electron microscopy (TEM)

Transmission electron microscopy evaluations were performed to visualize the internal morphological structure of optimized gliclazide loaded PLGA SGNCs formulation and its placebo formulation, more closely using TEM (Hitachi, HT7700 TEM, Tokyo, Japan) at an acceleration voltage of 80 kV and viewed at a magnification of 50,000x. Nanoparticles were diluted with distilled water and a drop of diluted sample was placed on a formvar-coated copper grid, and after complete drying, the sample was focused on a layer of photographic film grid and images were captured^63^^.^

### Atomic force microscopy (AFM)

AFM is a technique widely used to characterize the topographic geometry properties of nanoparticles. The topographic geometry properties such as two and three-dimensional view of atomic force microscopy images of optimized gliclazide loaded PLGA SGNCs formulation and placebo were further characterized by AFM (Park system, XE-70). A drop (5 µl) of optimized nanocrystal formulation and its placebo suspension were placed on a mica sheet and allowed to air dry for 5–10 min. The sample was further mounted on the microscope scanner. The shape was observed and imaged in ACAFM mode with frequency 166.6 kHz and scan speed 0.502 µm/s^61^.

### X-ray diffraction study

X-ray diffraction patterns and degree of crystallinity of pure gliclazide and gliclazide loaded PLGA SGNCs were measured in an X-ray diffractometer (PANalytical, X’Pert PRO MPD PW 3040/60). The measurements were performed at 2θ diffraction angles from 2° to 50° range using CuKα radiation (45 kV, 40 mA) as the X-ray source and the rate of scanning was 1° min^−1^^ 61,63^.

### Differential scanning calorimetric (DSC) study

The thermal behavior and physicochemical drug excipient compatibility between pure gliclazide and polymers were evaluated using a differential scanning calorimeter (DSC 8500, PerkinElmer, São Paulo, Brazil). The samples of pure gliclazide, physical mixture of pure gliclazide with PLGA, poloxamer 188 and optimized gliclazide loaded PLGA SGNCs formulation were accurately weighed, crimped and sealed in a standard aluminum pan heated over a temperature range from 0 to 250 °C, at a constantly increasing scanning rate of 15 °C/min. The thermal scanning of samples was carried out in the oven by purging nitrogen gas at a flow rate of 20 ml/min^63,64^.

### Fourier transform infrared (FTIR) spectroscopy

In the FTIR spectroscopic study, pure gliclazide, PLGA, poloxamer 188, physical mixture of GLZ with the excipients and lyophilized optimized gliclazide loaded PLGA SGNCs formulation were scanned over wavenumber 4000–400cm^−1^ by KBr pellet method in an inert environment using a Perkin Elmer Spectrum 100 FT-IR spectrometer (Perkin Elmer Inc., Wellesley, MA, USA). The IR spectral analysis was carried out to understand the drug excipients compatibility and possible interaction existing in the development of second generation nanocrystal formulation^61,64^.

### *In vitro* drug release studies

The *in vitro* drug release studies were performed for pure gliclazide and optimized gliclazide loaded PLGA SGNCs formulation for 72 h by using a dialysis system. Each dialysis bag (VIKING dialysis tubing, molecular weight cutoff 12 000 Da, pore size: 25 Å) was loaded with 2 ml of sample which was then sealed and immersed in 200 mL of medium (0.1 N hydrochloric acid pH 1.2, phosphate buffer solution pH 6.8) at 37 °C under 100 rpm continuous stirring. At predetermined intervals, 5 ml of dissolution medium was taken from outside the dialysis bag and replaced with a fresh 5 ml dissolution medium to maintain the sink condition. The amount of drug released was analyzed by a UV spectrophotometer (UV–1900 spectrophotometer, Shimadzu, Tokyo, Japan) at 229 nm. The drug release behavior of the pure gliclazide and optimized SGNCs formulation were compared and characterized. All measurements were performed in triplicate with similar experimental conditions^62^.

### *In vivo* pharmacokinetic-pharmacodynamic study

Healthy male albino rats (Sprague-Dawley) with average weight 200 ± 20 g, were obtained from Chennur Suppliers, Malaysia and housed in clean polypropylene cages, maintained under standard environment conditions, controlled temperature (25 °C) with a 12/12-h day-night cycle and were fed with standard rat feed and free access of water ad libitum. The animals were acclimatized for two weeks before the initiation of the experiment. The animal experimental protocol was evaluated in accordance with the institutional animal ethical committee guidelines for the care and use of laboratory animals and approved by IMU Joint Committee on Research & Ethics, International Medical University, Malaysia (Ethics Committee/IRB Ref. No: 4.19/JCM-170/2018)^62,63^.

### Induction of diabetes

The SD rats were fasted overnight before the induction of type 2 diabetes by administering a single dose of Streptozotocin (STZ) and nicotinamide (NAD). Administration of STZ and NAD reduce the β-cells, decrease pancreatic insulin stores and impaired insulin secretion leads to hyperglycemia condition in SD rats, which serves as a model for type 2 diabetes for the experimental studies. Streptozotocin single intravenous injection (80 mg/kg) prepared in citrate buffer pH 4.5 was administered followed by, nicotinamide (210 mg/kg, i.p.) prepared in normal saline administered 15 min later to the rats. The STZ-NA induced type 2 diabetic rats are prone to hypoglycemia and hence 6 h after induction, 20% of glucose solution was administered to the SD rats for the next 24 h. The rats were monitored by their weight, polydipsia, and mobility throughout the study. On the 7th day of STZ-NA administration, random blood glucose level was measured and the rats with blood glucose level within 200 mg/dl to 600 mg/dl was confirmed with type-II diabetes, which were used for a further research study^43,44,65^.

The experimental rats were divided into four groups, each comprising six rats:

Group I: Rats treated with citrate buffer (Normal control)

Group II: STZ-NA -induced diabetic rats that were treated with pure gliclazide at a single dose of 2.5 mg/kg via oral administration

Group III: STZ-NA -induced diabetic rats treated with placebo

Group IV: STZ-NA -induced diabetic rats treated with optimized gliclazide loaded PLGA SGNCs formulation at a single dose of 2.5 mg/kg via oral administration

### Sample preparation for HPLC analysis

A modified high-performance liquid chromatography (HPLC) bioanalytical method was adopted for the estimation of plasma gliclazide concentration of rats. In brief, 10 µl plasma was added to 50 µl of internal standard working solution (glibenclamide, 100 ppm) and vortex mixed for 3 min. Acetonitrile was added to the plasma samples in a 2:1 ratio and after vortexing for 5 min and centrifuged at 10,000 rpm for 5 min; the supernatant was withdrawn and dried using nitrogen evaporator. The residue was reconstituted with 200 µl of mobile phase and 20 µl was injected into the HPLC column, which consisted of a Flexar HPLC system (PerkinElmer, Waltham, MA, USA), a C18 analytical column (150 × 4.6 mm id, 5 µm particle size, Brownlee Analytical) and UV-Visible detector (PerkinElmer) set at 229 nm. The mobile phase was acetonitrile 49% and HPLC grade water of pH 6.8 (H20Pro-UV-T,water System, Arium pro, Sartorius) 51% at a flow rate of 1 ml/min. Under these conditions, the retention time for gliclazide was 5.45 min. A gliclazide calibration curve with a linear regression coefficient (r2) value 0.9963 was constructed and used in the drug bioanalytical analysis^66,67^.

### Pharmacodynamic study

A comparative pharmacodynamic response study of pure gliclazide, optimized gliclazide SGNCs, and placebo formulation were carried out using STZ-NA induced type-II diabetes rat model. Blood samples were collected from retro-orbital plexus of SD rats under ether anesthesia at regular intervals for 24 h at different time points (0, 0.5, 1, 2, 4, 8, 12, 24 h) after oral administration of pure gliclazide, optimized gliclazide loaded PLGA SGNCs and placebo formulation. Blood samples for the normal control (non-diabetic rats) and diabetes-induced SD rats were collected and blood glucose concentration was determined using glucometer (Accu-Check Active, Roche) by placing a drop of blood on the glucose strip (Accu-Check Active, Roche)^62,63^.

### Pharmacokinetic and bioavailability study

Pharmacokinetic and bioavailability study of pure gliclazide and optimized SGNCs formulation were performed in diabetes-induced SD rat model. For each SD rat, retro-orbital plexus under ether anesthesia, blood samples were collected at predetermined time points such as 0, 0.5, 1, 2, 4, 8, 12, 24 h. To isolate plasma, blood samples were centrifuged at 13,000 rpm for 5 min. The collected plasma sample was kept at −20 °C until further analysis. The plasma gliclazide concentration was estimated using a HPLC bioanalytical method. The gliclazide pharmacokinetic parameters and bioavailability data were calculated based on gliclazide plasma concentration versus time curves of non-compartmental analysis employing kinetic 5.1 version software (Adopt scientific). The area under the curve (AUC) was calculated using the trapezoidal method. The derived pharmacokinetic parameters and bioavailability data were further statistically analyzed by SPSS software (v 12.0; SPSS, Inc, Chicago, IL)^62,63^.

### Pharmacokinetic - pharmacodynamic correlation study

A pharmacokinetic and pharmacodynamic correlation was established to study the effect of nanonization of second-generation nanocrystals of gliclazide over pure gliclazide with respect to blood glucose lowering capacity. Plasma gliclazide concentration obtained from pharmacokinetic study and plasma glucose level of pharmacodynamic study of pure gliclazide, optimized gliclazide loaded PLGA SGNCs and placebo formulations were performed by point to point correlation approach^68^.

### Stability study

To investigate the physicochemical stability, a short-term stability study for optimized gliclazide loaded PLGA SGNCs formulation was carried out at three different temperatures (4 °C, 25 °C and 40 °C) for 30 days. Gliclazide loaded PLGA SGNCs formulation was equally divided into three vials and stored in a sealed condition at a designated temperature environment. Particle size, zeta potential, and polydispersity index of optimized gliclazide loaded PLGA SGNCs formulation were measured at different sampling time points, day 0 (formulation day), day 3, day 7, day 14 and day 30^62,69^.

### Statistical analysis

All data are expressed as mean ± standard deviation, wherever applicable of n = 3. To analyze the differences for the animal studies, a paired Student’s t-test was performed. The nanoformulations were compared by one-way ANOVA followed by Tukey’s test when equal variances are assumed, whilst Dunnett’s C test was applied when equal variances are not assumed.

## Supplementary information

Dataset 1

## Data Availability

All data generated or analysed during this study are included in this published article.
