# Supplementary material for: Fabrication of Second Generation Smarter PLGA Based Nanocrystal Carriers for Improvement of Drug Delivery and Therapeutic Efficacy of Gliclazide in Type-2 Diabetes Rat Model
Source: Sci Rep. 2019 Nov 22;9:17331. doi: 10.1038/s41598-019-53996-4 (PMC6874704; doi:10.1038/s41598-019-53996-4)
Supplement: Supplementary file 1 — Dataset 1 [file 41598_2019_53996_MOESM1_ESM.pdf]

Title page :

Fabrication of Second Generation Smarter PLGA Based Nanocrystal Carriers for Improvement of Drug Delivery and Therapeutic Efficacy of Gliclazide in Type-2 Diabetes Rat Model

Authors list:

Bibhu Prasad Panda [1]*, Rachna Krishnamoorthy [1], Subrat Kumar Bhattamisra [2]*, Naveen Kumar Hawala Shivashekaregowda [3], Low Bin Seng [4], Sujata Patnaik [5]

[1] Department of Pharmaceutical Technology, School of Pharmacy, Taylor's University, Lakeside Campus, No 1, Jalan Taylor's, 47500 Subang Jaya, Selangor, Malaysia.

[2] Department of Life Sciences, School of Pharmacy, International Medical University, Kuala Lumpur 57000 Malaysia.

[3] School of Pharmacy, Taylor's University, Lakeside Campus, No 1, Jalan Taylor's, 47500 Subang Jaya, Selangor, Malaysia.

[4] School of Medicine, Taylor's University, Lakeside Campus, No 1, Jalan Taylor's, 47500 Subang Jaya, Selangor, Malaysia.

[5] University College of Pharmaceutical Sciences, Kakatiya University, Warangal, Telangana, India.

Supplementary information

Sample	Blood glucose level (mg/dl)							
	0 h	0.5 h	1 h	2 h	4 h	8 h	12 h	24 h
Pure drug (API)	600 ± 67.1	471.7 ± 50.2	336 ± 35.6 [b]	254 ± 30.4 [a]	354.3 ± 40.4	371.3 ± 40.7 [a]	555.3 ± 35.7	391.3 ± 42.8 [b]
Diabetes control (Placebo)	600 ± 65.7	560 ± 58.9	566 ± 57.1	550.3 ± 60.5 [d]	568.7 ± 60.5	555.3 ± 67.3 [a]	470.7 ± 56.7	561.7 ± 62.1
SGNCF1	570.7 ± 60.7	252 ± 30.7 [b]	169 ± 20.9 [d]	112.3 ± 15.9 [d]	298.7 ± 34.2 [b]	265.3 ± 32.7 [b]	277 ± 34.8 [b]	172.7 ± 26.5 [d]

[a] $P<0.05$ vs. 0 h; [b] $P<0.01$ vs. 0 h ;SD: standard deviation; (n = 6).

Table 1. Responses of pure gliclazide, placebo and optimized formulation, SGNCF1 on blood glucose level in type II diabetic induced rats.

Storage time	Storage temperature (°C)	Average particle size (nm)	Zeta potential	Polydispersity index
Day 0	4	106.57 ± 1.43	-18.43 ± 0.61	0.22 ± 0.01
	25	107.90 ± 1.83	-18.97 ± 0.68	0.19 ± 0.06
	40	106.07 ± 1.86	-18.77 ± 0.83	0.19 ± 0.06
Day 3	4	106.80 ± 1.61	-18.10 ± 0.60	0.24 ± 0.01
	25	104.40 ± 1.44	-18.70 ± 0.70	0.22 ± 0.01
	40	107.73 ± 0.87	-17.13 ± 0.15	0.22 ± 0.01
Day 7	4	109.03 ± 2.61	-16.67 ± 0.42	0.24 ± 0.01
	25	109.47 ± 1.86	-16.77 ± 0.72	0.23 ± 0.01
	40	111.47 ± 2.00	-16.47 ± 0.74	0.24 ± 0.01
Day 14	4	107.60 ± 2.16	-15.73 ± 0.68	0.30 ± 0.02
	25	114.47 ± 3.95	-15.80 ± 0.96	0.29 ± 0.06
	40	116.80 ± 2.55	-15.23 ± 0.45	0.31 ± 0.04
Day 30	4	107.73 ± 2.83	-14.70 ± 0.87	0.33 ± 0.01
	25	110.87 ± 2.42	-14.50 ± 0.46	0.34 ± 0.03
	40	118.83 ± 3.19	-13.70 ± 0.26	0.37 ± 0.03

Table 2. Stability study for optimized gliclazide loaded PLGA SGNCs formulation.

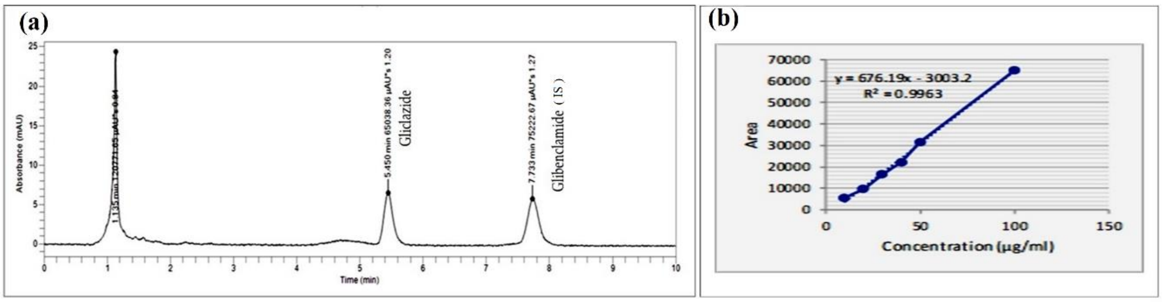

Figure 1. (a) HPLC chromatogram of plasma analysis containing 100 ug/ml gliclazide and 100 µg/ml internal standard (glibenclamide) at 229 nm; (b) calibration curve of gliclazid
